# Pars plana vitrectomy with/without encircling scleral band for treatment of retinal detachment associated with buphthalmos

**DOI:** 10.1186/s40942-021-00310-y

**Published:** 2021-05-13

**Authors:** Hammouda Hamdy Ghoraba, Sameh Mohamed El Gouhary, Ali Ahmed Ali Ghali, Mohamed Ahmed Abdelhafez, Adel Galal Zaky, Mahmoud Leila, Mohamed Amin Heikal, Hosam Othman Mansour

**Affiliations:** 1 Ophthalmology Department, Faculty of Medicine, Tanta University, Tanta, Egypt; 2 Magrabi Eye Hospital, Tanta, Egypt; 3 Ophthalmology Department, Faculty of Medicine, Menoufia University, Shibīn al-Kawm, Menoufia Egypt; 4 Ophthalmology Department, Faculty of Medicine, Al-Azhar University, Damietta Branch, New Damietta, Egypt; 5 Retina Department, Research Institute of Ophthalmology, Giza, Egypt; 6 Ophthalmology Department, Faculty of Medicine, Benha University, Banha, Egypt; 7 Magrabi Eye Hospital, Eastern Province, Kingdom of Saudi Arabia

**Keywords:** Retinal detachment, Buphthalmos, Globe survival after vitrectomy for buphthalmos

## Abstract

**Background:**

To evaluate the results of pars plana vitrectomy (PPV) and silicone oil (SO) tamponade with or without encircling scleral band for repair of rhegmatogenous retinal detachment (RRD) in children with buphthalmos.

**Patients and methods:**

Retrospective comparative nonrandomized interventional case series including consecutive patients who underwent PPV with or without encircling band and SO tamponade for RRD associated with buphthalmos.

**Results:**

The study included 19 eyes of 19 children. Mean age was 8 years, range 3–16 years. Mean follow-up period was 28 months, range 19–63 months. Globe survival has been achieved in 15 out of 19 eyes (79%). Phthisis bulbi was reported in four cases (22%). Eight patients (42%) achieved ambulatory vision. Most eyes initially achieved anatomical success.

**Conclusion:**

Despite the poor visual and anatomical results of RRD repair in eyes with buphthalmos, globe survival might be the rationale for surgery in such cases. Globe preservation could avoid the psychological and social consequences of phthisis bulbi in non-operated children.

## Introduction

Rhegmatogenous retinal detachment (RRD) is a major cause of loss of vision in adults. RRD occurs often in children. The reported incidence is 3–12% of all cases. Rarely, RRD is associated with primary congenital glaucoma [1–6]. The clinical features, underlying causes, outcome of surgical procedure and visual prognosis differ from those of adults. Surgical treatment poses major challenge to the vitreoretinal surgeon because children are more prone to develop macula-off RRD, proliferative vitreoretinopathy (PVR) and late presentation, hence longstanding retinal detachment. Consequently, the overall prognosis of surgery, particularly the visual outcome is highly-guarded [6–10]. Small-gauge PPV along with improved insight into the pathogenesis of RRD have considerably booted the surgical outcome in these complicated cases [7–11]. Globe survival in this category of RRD might be a significant end-point for surgical intervention by preserving the globe and by avoiding sequelae of phthisis bulbi in childhood, particularly if patients needed an artificial implant for cosmesis [6–14]. The aim of the present study is to evaluate the results of PPV for repair of RRD associated with buphthalmos in children.

## Patients and methods

This is a retrospective comparative interventional non-randomized case series that included children who had PPV for RRD associated with buphthalmos. Data of patients was retrieved from the medical records of all pediatric patients operated for RRD in a tertiary retinal center; Magrabi eye hospital, Tanta, Egypt, during the period from June 2008 to April 2019. Preoperative examination included; best-corrected visual acuity (BCVA) assessment and intraocular pressure (IOP) measurement when those were possible, slit-lamp examination, fundus examination using indirect ophthalmoscopy, and B-scan ultrasound evaluation.

### Surgical technique

A single surgeon (HG) operated on all cases under general anesthesia. An encircling scleral band style 240 (2.5 mm) was combined with PPV except in patients who had functioning filtering bleb, aqueous filtering devices, e.g. Ahmed’s valve, or presence of scleral ectasia. Pars plana lensectomy was performed in all phakic children without intra-ocular lens (IOL) implantation. PPV consisted of core vitrectomy; triamcinolone acetonide (TA)-assisted induction of posterior vitreous detachment (PVD). Perfluorocarbon liquid (PFC) stabilized the posterior retina during shaving the vitreous base. The surgeon then performed air-fluid exchange, endo-laser retinopexy to retinal breaks and in an encircling 360° pattern. Finally, SO/air exchange was done. The surgeon routinely used SO 5000 centistokes (cSt) in all patients. Inferior surgical iridectomy (at 6 O’clock) was created in all aphakic eyes. Sclerotomies were closed using 7/0 vicryl sutures.

### Postoperative care

Cooperative children were instructed to maintain prone position 8 h per day for 1 week. Those children had slit-lamp examination in the first postoperative day. If possible, fundus examination, and color fundus photography were done to asses and document retinal reattachment. The follow-up was scheduled at 1, 3, and 6 weeks, and then every 8 weeks. In each visit, we recorded BCVA and IOP when possible and assessed retinal re-attachment. Uncooperative or young children had examination under general anesthesia; where anterior segment was examined using the surgical microscope, IOP was measured using schiotz tonometer and the fundus was examined using the indirect ophthalmoscope.

The term globe survival refers to eyes that did not need enucleation until the end of the follow-up period. Main outcome measures were retinal reattachment, improvement of BCVA and globe survival rate.

### Management of recurrence

Patients who developed recurrent retinal detachment under SO during follow-up, had revision surgery that consisted of SO removal, TA-assisted removal of any residual vitreous cortex and peeling of epiretinal membranes. The surgeon performed relaxing retinotomy and/or retinectomy for immobile and shortened retinae when needed followed by air/fluid exchange, drainage of sub-retinal fluid, endolaser retinopexy and finally SO 5000 cSt injection and closure of sclerotomies using 7/0 vicryl sutures.

### Silicone oil removal

Silicone oil was removed in all eyes that had stable retinae for at least 6 months. The technique consisted of 2-port 20-gauge or 23-gauge sclerotomies or both combined). One port was used for infusion, and the other for SO aspiration. The retina was thoroughly examined looking at the retinal periphery and the site of retinal breaks. SO was extracted manually or aspirated followed by multiple partial air/fluid exchange. Sclerotomies were closed using 7/0 vicryl sutures. The parents of all children recruited for the study provided a written informed consent after receiving thorough explanation of all surgical procedures entailed, prognosis for vision, and possible complications.

## Results

### Baseline characteristics (shown in Tables 1, 2)

**Table 1:** Data of patients who underwent an encircling scleral band with PPV

Patients	Age (years)	Pre-operative visual acuity	Lens status	Retinal tear	Final retinal status	Corneal status	SOR	Postoperative visual acuity
1	9	HM	Pseudophakic	GT	Subtotal RD	Band keratopathy	No	HM
2	9	PL	Pseudophakic	GT	Attached	Clear	No	2/60
3	6	HM	Phakic	Undetected	Total RD	Band keratopathy	No	NPL
4	7	HM	Pseudophakic	superior tear	Attached	Clear	Yes	1/60
5	9	PL	Phakic	superior tear	Total RD	Band keratopathy	No	NPL
6	5	HM	Pseudophakic	superior tear	Attached	Clear	yes	1/60
7	4	HM	Pseudophakic	Undetected	Attached	Clear	No	HM
8	7	HM	Pseudophakic	GT	Attached	Clear	Yes	1/60

*GT* giant tear, *HM* hand movement, *PL* perception of light, *NPL* no perception of light, *RD* retinal detachment, *SOR* silicone oil removal

**Table 2:** Data of patients who underwent PPV without an encircling scleral band

Patients	Age (years)	Preoperative visual acuity	Lens status	Retinal tear	Final retinal status	SOR	Corneal status	Postoperative visual acuity
1	10	PL	Phakic	Multiple	Attached	No	Clear	NPL
2	8	PL	Phakic	GT	Total RD	No	Band keratopathy	HM
3	6	HM	Cataractous	Undetected	Attached	No	Clear	1/60
4	16	PL	Cataractous	Single	Attached	No	Clear	PL
5	10	HM	Aphakic	GT	Attached	No	Clear	1/60
6	11	HM	Cataractous	Multiple	Attached	Yes	Clear	HM
7	4	PL	Pseudophakic	Undetected	Total RD	No	Band keratopathy	NPL
8	3	HM	Phakic	GT	Attached	No	Clear	HM
9	10	CF	Phakic	Multiple	attached	No	Clear	2/60
10	9	PL	Subluxated	Single	Subtotal RD	No	Band keratopathy	NPL
11	4	HM	Pseudophakic	Undetected	Attached	No	Clear	1/60

*CF* counting fingers, *GT* giant tear, *HM* hand movement, *PL* perception of light, *NPL* no perception of light, *RD* retinal detachment, *SOR* silicone oil removal

The study recruited 19 eyes of 19 children. Male gender constituted 16 patients (84.2%). Mean age was 8.3 years, (range 3–16 years). At the time of recruitment, 18 children (94.7%) already had sub-scleral trabeculectomy (SST) and one child (5.3%) had glaucoma drainage device; Ahmed glaucoma valve done elsewhere. In terms of number and location of retinal breaks, 6 eyes (31.6%) had giant retinal tear (GRT), 3 eyes (15.8%) had multiple retinal tears; whereas 5 eyes (26.3%) had a single retinal break. We are unable to detect retinal breaks in 5 eyes (26.3%). An encircling scleral band was combined with PPV in 8 cases (42%). A scleral band was not applied in the remaining 11 cases. One eye had functioning Ahmed glaucoma valve, 6 eyes had scleral thinning and/or ectasia and 4 eyes had functioning bleb.

### Post-operative anatomical outcome

All cases had successful retinal reattachment after the first surgery. During the follow-up period, 4 patients (21%) had revision of surgery and silicone reinjection due to recurrent RD. By the end of follow-up period 6 eyes (31.6%) had recurrent RD under SO that was either localized or total. SO was removed after stability of retinal status for at least 6 months in 4 out of 19 cases (21%). SO was not removed in the remaining cases due to poor visual potential, hypotony or band shaped keratopathy, which prevented proper retinal evaluation. The rate of globe survival with free ocular movements in all directions of gaze was 78.9%.

Different degrees of exotropia developed in 6 eyes (31.6%). Four eyes (21%) developed phthisis bulbi at variable periods during follow-up.

#### Post-operative IOP

In 14 patients, IOP was controlled without anti-glaucoma drugs. IOP elevation developed after surgery in 5 patients, of which 2 patients required cyclophotocoagulation using diode laser and 3 patients had their IOP controlled using anti-glaucoma medications.

#### Corneal clarity

The cornea remained clear in 13 eyes. In 6 eyes SO migration to the anterior chamber with prolonged contact to the endothelium resulted in band keratopathy that contributed to their final poor visual outcome.

### Post-operative functional outcome

Post-operatively, 8 eyes (42%) achieved ambulatory vision, of which 2 eyes had final BCVA of 2/60 and 6 eyes had final BCVA of 1/60. Final BCVA remained stationary (HM or PL) in 6 eyes (31.5%). Vision deteriorated in 5 eyes (26%). Poor visual outcome occurred mainly due to band keratopathy, PVR, and glaucomatous optic atrophy.

## Discussion

Congenital glaucoma is a major cause of blindness in children, despite its low incidence (1:10.000 births). The disease is bilateral in approximately 75% of cases. Male patients account for approximately 65% of cases. Many authors corroborate the theory of polygenic pattern of inheritance [15]. In pediatric population, RRD associated with congenital glauc.ma comprises a few number of children treated for RRD. Hence, there is limited data for this group of patients in published literature. Gupta et al. [16] reported the higher incidence of peripheral retinal degenerations and recommended screening especially in eyes with axial length ≥ 26 mm. Many authors reported poor visual outcome in cases of RRD associated with buphthalmos, despite retinal reattachment [6, 12, 13]. Satofuka and his colleagues reported poor visual outcome after management of RRD associated with buphthalmos in three eyes of three consecutive patients, despite retinal reattachment in all cases after PPV. In comparison, in the present study 8 eyes (42%) achieved ambulatory vision. Al-Harthi et al. [13] retrospectively reviewed 34 cases of RD in eyes with buphthalmos between 1982 and 2003. Twenty-four eyes underwent surgical repair (vitrectomy and/or scleral buckle with gas or silicone oil tamponade). The authors reported recurrent RD in 37.5% of eyes within a mean 3-year follow-up period, compared to 6 out of 19 eyes (31.5%) in our study. At the end of follow-up period, 2 eyes had BCVA of 20/200; the rest of cases had poor vision ranged form of hand movements to LP, and NLP. They concluded that the poor prognosis was related to late diagnosis of RD in children, corneal scarring, pre or postoperative PVR and glaucomatous optic atrophy. Our findings in the present study were congruous with the latter conclusion of these authors. Despite that the fore-mentioned studies provided evidence of unfavorable visual outcome of vitrectomy to repair RRD associated with buphthalmos, our results suggest that globe preservation might offer sound rationale for vitrectomy to repair RD in these eyes. It seems wise to preserve the globe as much time as possible and to avoid phthisis bulbi with all its adverse sequelae at this age. The issue of globe preservation in cases of pediatric RD was discussed by Read et al. [14]; the authors achieved globe preservation with partial retinal re-attachment after RD surgery in 77% of a subgroup of patients with traumatic retinal detachment. The authors performed enucleation in 3 out of 60 eyes (5%), whereas phthisis bulbi developed in 11 out of 60 cases (18%). In a subgroup of patients with RD, the authors achieved globe preservation in 69% of eyes. They performed enucleation in 4 out of 67 eyes (5.8%), phthisis bulbi developed in 17 out of 67 cases (25.37%). In eyes with RRD cases, they reported globe preservation in 86% of eyes, enucleation in 4 out of 51 eyes (4%), and phthisis in 5 out of 51 of eyes bulbi (9.8%. In comparison globe survival was achieved in 15 of 19 cases (79%) in our study, with ambulatory vision in 8 out of 19 eyes (42%). Cooling et al. [14] concluded that retinal detachment in eyes with buphthalmos was a highly complicated scenario and carries a very poor visual prognosis. Retinal detachment surgery was done in 13 eyes of 12 patients. Globe survival was the main benefit of surgery. The authors reported retinal re-attachment in 3 eyes only for 6 months or longer and anatomical reattachment with visual improvement in 2 of the 5 eyes which had SO injection [12]. The results and conclusions of that study corroborate our rationale for this surgery. An important limitation of the study is the small sample size recruited due to the rarity of the condition studied. Another limitation is the retrospective nature of the study that relied on data collection form medical records from different glaucoma clinics with subsequent discrepancies in data compilation.

## Conclusion

Repair of retinal detachment in eyes with buphthalmos carries poor visual and anatomical results, thus globe preservation might be the rationale of surgery in such cases. Surgical repair and globe preservation conservation could avoid the psychological and social consequences of phthisis bulbi in non-operated eyes. Moreover, ambulatory vision might be obtained in some eyes.

## Data Availability

All the study data and relevant materials are available on reasonable request from the corresponding author.
